# Asclepius and Yellow Ribbon techniques: Efficacious alternative strategies for advancing a coronary sinus electrophysiology catheter

**DOI:** 10.1111/anec.12740

**Published:** 2020-01-20

**Authors:** Tse‐Wei Chen, Mu‐Shiang Huang, Wei‐Da Lu, Yu‐Hao Wu, Ju‐Yi Chen

**Affiliations:** ^1^ Division of Cardiology Department of Internal Medicine National Cheng Kung University Hospital College of Medicine National Cheng Kung University Tainan Taiwan

**Keywords:** coronary sinus, electrophysiology

## Abstract

**Background:**

Inserting an electrophysiological (EP) catheter into the coronary sinus (CS) via the femoral vein can be difficult and time‐consuming in patients with variants of the CS orifice or lumen curve. Our experience with such patients inspired us to develop two new techniques: the Asclepius and Yellow Ribbon techniques.

**Methods:**

Data from a 4‐year period were retrieved from records of patients undergoing radiofrequency ablation for paroxysmal supraventricular tachycardia (PSVT) or Wolff–Parkinson–White (WPW) syndrome. Data were analyzed to determine the success and complication rates of conventional and alternative techniques for catheter placement.

**Results:**

The success rate of the Asclepius technique was 96.7% (30/31) and that of the Yellow Ribbon technique was 100.0% (7/7). The overall success rate of these two techniques was 97.3% (37/38).

**Conclusions:**

With a high success rate, shorter procedure time, and no complications, the Asclepius and Yellow ribbon techniques may be safe, inexpensive, and effective alternative strategies for EP catheter placement in patients with difficult coronary sinus orifice access.

## 1 INTRODUCTION

The detection and recording of electrical signals is essential to the assessment of cardiac conduction for diagnosing cardiac dysfunction and planning treatment. Multi‐electrode catheters are routinely used in clinical electrophysiological (EP) assessments. To assess the coronary sinus (CS) (the vein situated between the left atrium and left ventricle), a decapolar catheter is advanced through a central venous access. In our hospital, the catheter is typically inserted via the femoral vein rather than the internal jugular or subclavian vein to avoid complications such as pneumothorax or neck hematoma with airway compromise (Eisen et al., 2006; Parienti et al., 2015). Femoral access is also more acceptable to patients who are nervous about the neck insertion approach.

Occasionally, we encounter challenging variations in CS anatomy related to the vessel size, orifice direction, or lumen curvature (Mak, Hill, Moisiuc, & Krishnan, 2009; Mlynarski, Mlynarska, Tendera, & Sosnowski, 2011). These variants make advancement of the EP catheter tip more difficult owing to the fixed curve of the decapolar catheter. Difficulties in catheter advancement lead to prolonged operation time and greater radiation exposure. An alternative method involves the use of a steerable decapolar EP catheter. This device has ergonomic handling and a rotary dial designed for fine tip movements that allow for catheter advancement through challenging anatomy (Er, Yuksel, Hellmich, & Gassanov, 2015; Manolis, Koulouris, & Tsiachris, 2018). However, the steerable EP catheter is expensive, costing 17,189 New Taiwan Dollars (557 USD), 40% more than the fixed‐curve decapolar EP catheter. As an alternative, we have developed two innovative, safe, and highly successful procedures, the “Asclepius technique” and “Yellow ribbon technique,” for advancing the standard EP catheter into the CS.

## 2 METHODS

### 2.1 Subjects

**Figure 1:**
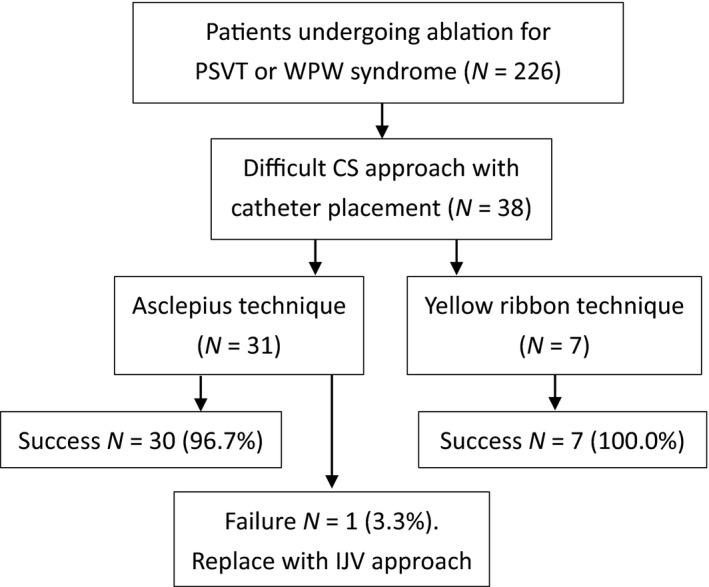
Algorithm for data recruitment. CS, coronary sinus; IJV, internal jugular vein; PSVT, paroxysmal supraventricular tachycardia; WPW, Wolff–Parkinson–White

This study was performed in a tertiary care center. We reviewed a total of 226 cases in 4 years from August 1, 2015 to July 31, 2019 involving catheter radiofrequency ablation in patients with paroxysmal supraventricular tachycardia (PSVT) or Wolff–Parkinson–White (WPW) syndrome. Difficulty was encountered with the CS approach in 38 of these cases (16.8%), prompting application of the Asclepius or Yellow Ribbon technique (Figure 1). We compared the characteristics and outcomes between patients undergoing conventional and alternative insertion procedures (Table 1).

**Table 1:** Comparison of patient characteristics between those undergoing conventional and alternative insertion procedures

	Insertion Method		*p* value
	Alternative (*n* = 38)	Conventional (*n* = 188)	
Age	48 ± 19	44 ± 20	.31
AVNRT	24 (63.1%	123 (65.4%)	.78
AVRT, right kent	5 (13.1%)	25 (13.2%)	.98
AVRT, left kent	9 (23.6%)	40 (21.2%)	.74
Gender (Male)	14 (36.8%)	68 (36.1%)	.93
DM	6 (15.7%)	16 (8.5%)	.16
Hypertension	13 (34.2%)	38 (20.2%)	.06
Hyperlipidemia	8 (21.0%)	33 (17.5%)	.61
CKD	2 (5.2%)	11 (5.8%)	.88
Paroxysmal AF	4 (10.5%)	7 (3.7%)	.07
CHF	1 (2.6%)	4 (2.1%)	.84
Stroke	1 (2.6%)	1 (0.5%)	.20

Abbreviations: AF, atrial fibrillation; AVNRT, atrioventricular nodal reentrant tachycardia; AVRT, atrioventricular reentrant tachycardia; CHF, congestive heart failure; CKD, chronic kidney disease; DM, diabetes mellitus.

### 2.2 Asclepius technique

**Figure 2:**
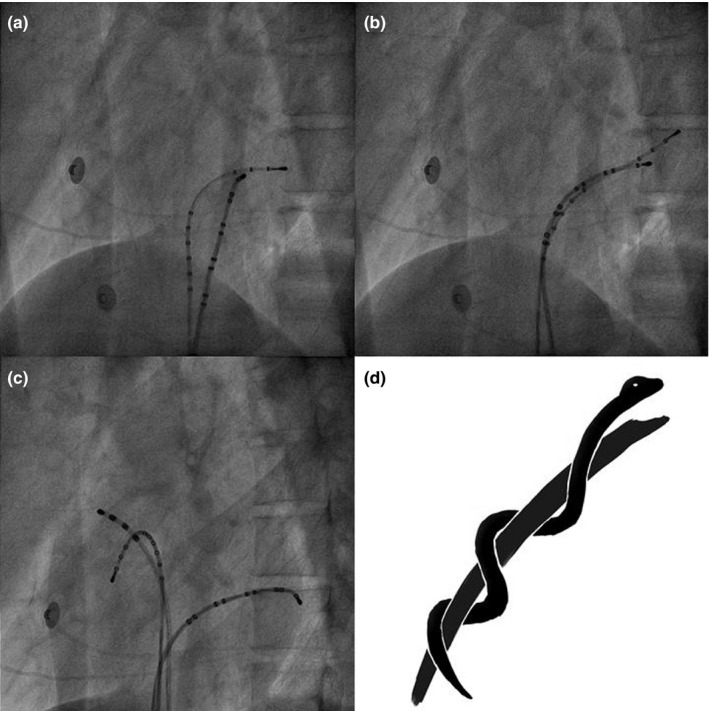
Photographs and drawings illustrating the steps of the Asclepius technique. The angles of the fluoroscopy are 60 degree straight LAO. (a) Engagement of the HIV‐RV steerable EP catheter into the CS. (b) Advancement of the CS fixed‐curve EP catheter, winding around and up into the CS. (c) Successful CS cannulation and final lead position. (d) Winding feature of the Asclepius technique

Our newly developed Asclepius technique begins with the preparation of a fixed‐curve decapolar EP catheter (French gauge 6) (Response, Abbot Laboratories, Chicago, IL, USA) for the CS, a fixed‐curve quadrapolar EP catheter (French gauge 6) (Response, Abbot Laboratories) for the right atrium, and a steerable decapolar EP catheter (French gauge 6) (Livewire, Abbot Laboratories) for the HIS‐right ventricle (HIS‐RV). The angles of the fluoroscopy are 60 degree straight LAO. First, the HIS‐RV steerable catheter was introduced into the CS. Once in position, we place the fixed‐curve decapolar EP catheter with the tip toward the orifice of the CS. This second catheter then winded around the steerable catheter and up into the CS smoothly and quickly, resembling an Asclepius—the medical symbol depicting a snake winding around a staff (Figure 2). Finally, we withdrew the steerable catheter from the CS and back into the HIS‐RV (Video S1). The entire procedure takes no more than 5 min.

### 2.3 Yellow Ribbon technique

**Figure 3:**
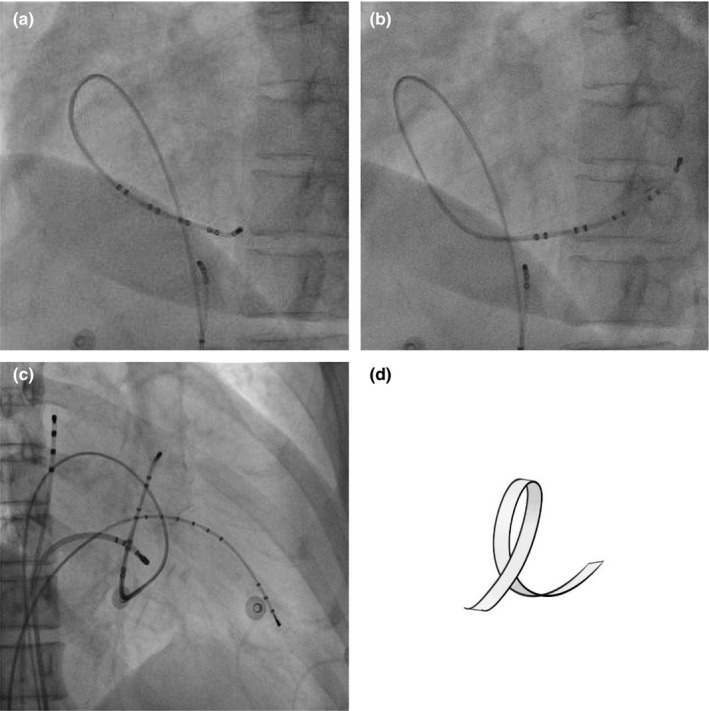
Photographs and drawings illustrating the steps of the Yellow ribbon technique. The angles of the fluoroscopy are 60 degree straight LAO. (a) Circumferentiation of the fixed‐curve EP catheter. (b) Advancement of the CS fixed‐curve EP catheter into the CS. (c) Successful CS cannulation and final lead position. (d) Circular feature of the Yellow ribbon technique

The fixed‐curve decapolar EP catheter was placed with the tip near the orifice of the CS. The catheter was then advanced around the right atrial chamber in a circle, until the tip, headed toward the end of the circle, spontaneously entered the CS. The shape of the tip movement through the process resembles a yellow ribbon (Figure 3). The angles of the fluoroscopy are also 60 degree straight LAO. This procedure proceeds quickly and smoothly (Video S2).

Basically, we perform the Asclepius technique at first for difficulty in CS placement. If prolonged attempt over 5 min, we do the Yellow ribbon technique subsequently. In rare cases, we just switch the techniques to each other not in absolute order of priority. In conclusion, no more than 5 min in each techniques were done by our experience. The supplementary video shows how each technique works.

### 2.4 Statistical methods

Continuous variables are presented as the mean ± standard deviation (*SD*). The chi‐squared test was used for categorical variables, and the independent samples Student's *t* test was used for continuous variables. All statistical analysis was carried out using SPSS 23 for Windows (IBM Corp.).

## 3 RESULTS

Alternative techniques were needed to insert the catheters in 38 of the patients. No significant differences in baseline characteristics were observed between those treated using alternative and conventional techniques, except that more patients with hypertension and paroxysmal atrial fibrillation were included in the alternative technique group. The Asclepius technique was used in 31 of the 38 patients undergoing an alternative technique. Of these patients, 30 (96.7%) were successful. Only one attempt failed, as the steerable catheter would not engage. This failure was followed by successful recannulation with a different catheter using the curve‐shaped guidance of a new central access on the right internal jugular vein. The remaining 7 patients were treated using the Yellow ribbon technique, with 100% success and no complications. The overall success rate for patients undergoing alternative techniques was 97.3% (37/38).

## 4 DISCUSSION

The present study shows that our newly developed Asclepius and Yellow Ribbon techniques yielded impressively high success rates (Asclepius, 96.7%; Yellow Ribbon, 100%) for difficult CS catheter placement. No complications occurred. No patient required further neck internal jugular vein or thoracic subclavian venipuncture, so patient suffering and risk of complications were decreased.

For HIS bundle signal recording in Asclepius technique, the only steerable catheter is targeted to RV‐HIS originally. And the nonsteerable CS catheter we used is designed for CS signal, not available for RV‐HIS. Therefore, we have to exchange catheters step‐by‐step: (a) The steerable RV‐HIS catheter to CS. (b) The nonsteerable CS catheter to CS by the Asclepius technique. (c) Pullback the RV‐HIS catheter from CS and advance to RV‐HIS in position.

We attribute the success of the Asclepius technique to several factors. First, the steerable catheter provides better handling of the distal tip movement. Second, after the steerable catheter engages into position with the orifice and modified lumen of the CS, the fixed‐curve catheter can pass through. Third, placement of the steerable catheter in situ may lower the resistance of the tract to help the fixed‐curve catheter move more easily and deeper into the vessel.

In the Yellow ribbon technique, the circumferential shape of the catheter tip movement returns the tip back to the starting point near the CS orifice, providing a better curve that takes shape naturally and strong support. As in the ancient Chinese martial art Tai Chi, taking advantage of the internal leverage of circular motion makes a forward pushing motion easier.

## 5 LIMITATIONS

Theoretically speaking, those patients with right atrial dilation or enlargement are difficult for engagement of the CS to a certain extent, by using nonsteerable CS catheter as the original method. One of limitations in our retrospective study is lack of robust formal echocardiography report in each patients received the procedures. We just prove and assure that these new techniques provide a better cost‐performance way to overcome some structural heart diseases such as atrial dilation or enlargement.

According to the rare case failure to apply such techniques, the possible causes lead to unworkable including extreme chamber size with CS orifice in sharp angle, anomaly of CS opening, or operator's technical experience.

## 6 CONCLUSIONS

The Asclepius and Yellow Ribbon techniques are safe, cost‐effective, and highly successful alternative strategies to facilitate catheter placement for electrophysiological assessments.

## CONFLICT OF INTEREST

The authors would like to thank the Ministry of Science and Technology of the Republic of China, Taiwan for financial support of this research under contract MOST 108‐2218‐E‐006‐019.

## AUTHOR CONTRIBUTION

Conception and design: J‐YC; data acquisition: T‐WC, Y‐HW; data analysis and interpretation: J‐YC, T‐WC, M‐SH, W‐DL; statistical analysis: J‐YC, T‐WC, M‐SH; drafting and finalizing the article: J‐YC, TWC, M‐SH; critical revision of the article for important intellectual content: J‐YC, M‐SH.

## ETHICS

This study was approved by the ethics committee of National Cheng Kung University Hospital and was conducted according to the guidelines of the International Conference on Harmonization for Good Clinical Practice.

## References

[anec12740-bib-0001] Eisen, L. A. , Narasimhan, M. , Berger, J. S. , Mayo, P. H. , Rosen, M. J. , & Schneider, R. F. (2006). Mechanical complications of central venous catheters. Journal of Intensive Care Medicine, 21(1), 40–46. 16698743 10.1177/0885066605280884

[anec12740-bib-0002] Er, F. , Yuksel, D. , Hellmich, M. , & Gassanov, N. (2015). Comparison of Conventional versus Steerable‐Catheter Guided Coronary Sinus Lead Positioning in Patients Undergoing Cardiac Resynchronization Device Implantation. PLoS ONE, 10(11), e0143292 10.1371/journal.pone.0143292 26599637 PMC4658090

[anec12740-bib-0003] Mak, G. S. , Hill, A. J. , Moisiuc, F. , & Krishnan, S. C. (2009). Variations in Thebesian valve anatomy and coronary sinus ostium: Implications for invasive electrophysiology procedures. Europace, 11(9), 1188–1192. 10.1093/europace/eup179 19587062

[anec12740-bib-0004] Manolis, A. S. , Koulouris, S. , & Tsiachris, D. (2018). Electrophysiology Catheter‐Facilitated coronary sinus cannulation and implantation of cardiac resynchronization therapy systems. Hellenic Journal of Cardiology, 59(1), 26–33. 10.1016/j.hjc.2017.07.008 28778735

[anec12740-bib-0005] Mlynarski, R. , Mlynarska, A. , Tendera, M. , & Sosnowski, M. (2011). Coronary sinus ostium: The key structure in the heart's anatomy from the electrophysiologist's point of view. Heart and Vessels, 26(4), 449–456. 10.1007/s00380-010-0075-3 21240507

[anec12740-bib-0006] Parienti, J.‐J. , Mongardon, N. , Mégarbane, B. , Mira, J.‐P. , Kalfon, P. , Gros, A. , … du Cheyron, D. (2015). Intravascular complications of central venous catheterization by insertion site. New England Journal of Medicine, 373(13), 1220–1229. 10.1056/NEJMoa1500964 26398070

